# Genome-Wide Identification, Evolution, and Co-expression Network Analysis of Mitogen-Activated Protein Kinase Kinase Kinases in *Brachypodium distachyon*

**DOI:** 10.3389/fpls.2016.01400

**Published:** 2016-09-16

**Authors:** Kewei Feng, Fuyan Liu, Jinwei Zou, Guangwei Xing, Pingchuan Deng, Weining Song, Wei Tong, Xiaojun Nie

**Affiliations:** ^1^College of Agronomy, Northwest A&F UniversityYangling, China; ^2^State Key Laboratory of Crop Stress Biology in Arid Areas, Northwest A&F UniversityYangling, China; ^3^Australia-China Joint Research Centre for Abiotic and Biotic Stress Management in Agriculture, Horticulture and Forestry, Northwest A&F UniversityYangling, China

**Keywords:** *Brachypodium distachyon*, co-expression network, expression profiles, MAPKKK family, MAPK cascades, signal pathway

## Abstract

Mitogen-activated protein kinase (MAPK) cascades are the conserved and universal signal transduction modules in all eukaryotes, which play the vital roles in plant growth, development, and in response to multiple stresses. In this study, we used bioinformatics methods to identify 86 MAPKKK protein encoded by 73 MAPKKK genes in *Brachypodium*. Phylogenetic analysis of MAPKKK family from *Arabidopsis*, rice, and *Brachypodium* has classified them into three subfamilies, of which 28 belonged to MEKK, 52 to Raf, and 6 to ZIK subfamily, respectively. Conserved protein motif, exon-intron organization, and splicing intron phase in kinase domains supported the evolutionary relationships inferred from the phylogenetic analysis. And gene duplication analysis suggested the chromosomal segment duplication happened before the divergence of the rice and *Brachypodium*, while all of three tandem duplicated gene pairs happened after their divergence. We further demonstrated that the MAPKKKs have evolved under strong purifying selection, implying the conservation of them. The splicing transcripts expression analysis showed that the splicesome translating longest protein tended to be adopted. Furthermore, the expression analysis of BdMAPKKKs in different organs and development stages as well as heat, virus and drought stresses revealed that the MAPKKK genes were involved in various signaling pathways. And the circadian analysis suggested there were 41 MAPKKK genes in *Brachypodium* showing cycled expression in at least one condition, of which seven MAPKKK genes expressed in all conditions and the promoter analysis indicated these genes possessed many cis-acting regulatory elements involved in circadian and light response. Finally, the co-expression network of MAPK, MAPKK, and MAPKKK in *Brachypodium* was constructed using 144 microarray and RNA-seq datasets, and ten potential MAPK cascades pathway were predicted. To conclude, our study provided the important information for evolutionary and functional characterization of MAPKKK family in *Brachypodium*, which will facilitate the functional analysis of BdMAPKKK genes, and also will facilitate better understanding the MAPK signal pathway in *Brachypodium* and beyond.

## Introduction

Mitogen-activated protein kinase (MAPK) cascades are highly conserved signal transduction pathways in eukaryotes, which included three main members of MAPK kinase kinases (MAPKKK or MEKK) and MAPK kinases (MKK or MEK) as well as MAPK (MPK; Nishihama et al., 1995). They achieve the functions by sequentially being phosphorylated. Upstream signals firstly activate the MAPKKKs, which in turn the MAPKKKs activate the MAPKKs by phosphorylating its motif of S/T-X_5_-S/T and then specific MAPKs as terminal components of the cascades are activated by MAPKKs via the phosphorylation of conserved motif TxY (MAPK Group, 2002; Rodriguez et al., 2010). Eventually, activated MAPKs phosphorylate various transcription factors, enzymes, or other signaling components to modulate the expression of downstream genes to complete signal amplification (Fiil et al., 2010; Rodriguez et al., 2010). Nowadays, many functions of MAPK cascades are found in plant involved in cell division, growth, and differentiation (Takahashi et al., 2010; Zhao et al., 2013), hormone response (Kieber et al., 1993; Yue et al., 2012; Wang et al., 2014), plant immunity (Asai et al., 2002; Mithoe, 2013), biotic and abiotic stress (Frye et al., 2001; Munnik and Meijer, 2001; Jammes et al., 2009; Kumar and Sinha, 2013; Shitamichi et al., 2013; Jiang et al., 2015; Çakır and Kılıçkaya, 2015).

Although the three members in MAPK cascades (MPKs, MKKs, and MAPKKKs) have been systematic investigated in *Arabidopsis*, rice, maize, cotton, soybean, and so on, only a few kinase-activation processes and signal networks have been illustrated clearly at present, of which most studies are limited in *Arabidopsis* (Janitza et al., 2012; Takáč and Šamaj, 2015). MEKK1-MKK4/5-MPK3/6-WRKY22/WRKY29/FRK1 cascade played an important role in *Arabidopsis* innate immunity, which was the first clarified MAPK signal pathway (Asai et al., 2002; Pitzschke et al., 2009). EKK1-MKK1/MKK2-MPK4/6-MKS1/WRKY33 has demonstrated in mediating cold and salt stress (Kovtun et al., 1998) and regulating innate immunity (Gao et al., 2008; Kong et al., 2012). ANP2/3-MKK6-MPK4/11/13 played role in the regulation of cytokinesis (Takahashi et al., 2010; Zeng et al., 2011); YODA-MKK4/5-MPK3/6 cascade negatively regulated the stomatal development (Wang et al., 2007). ANP1-MKK4/5-MPK3/6 participated in the regulation of reactive oxygen (Lee and Ellis, 2007). In addition, Raf5 (At1g73660) was confirmed to regulate the sugar resistant seedling development in *Arabidopsis* (Huang et al., 2014); *Raf12* (*MAP3K*δ*4*, At4g23050) was found to play an important role in ABA signaling by over-expression analysis (Shitamichi et al., 2013).

*Brachypodium distachyon* belongs to Pooideae subfamily and has a close genetic relationship with grain crop, including wheat, barley, and rye, which provide the major food source of human nutrition (Vogel et al., 2006). *Brachypodium* as a small grass, possesses many characteristics such as short generation time, self-pollination, a small genome size of 272 Mb (International Brachypodium Initiative, 2010), strong reproduction without fertile soil and easy genetic transformation, which make it become an attractive model for functional genomics in Pooideae (Draper et al., 2001; Brkljacic et al., 2011). Thus, revealing the regulatory network and signal pathway of MAPK cascade kinases in *Brachypodium* will provide the vital reference information for further functional analysis of MAPK cascade genes across wheat and barley.

Up to now, several studies have been conducted to investigate the mitogen-activated protein kinase cascades in *Brachypodium*. Chen et al. (2012) systematically identified and analyzed the BdMAPK and BdMAPKK gene family at genome-wide level. Jiang et al. (2015) identified the MAPKKK gene family in *Brachypodium* and comparatively analyzed the BdMAPKs, BdMAPKKs, and BdMAPKKKs to reveal the evolution and regulatory network of MAPK cascades in Brachypodium. However, a systematical investigation of the structural, evolutionary, and expressional characteristics of all the members of BdMAPKKKs, especially the expression profiles in circadian rhythm has not been performed, which limit the further study of this important signal pathway. In this study, an *in-silico* search of *Brachypodium* genome database was conducted to identify members of the *Brachypodium* MAPKKK gene family. A total of 86 MAPKKKs encoded by 73 genes were identified. A phylogenetic tree was constructed and the MAPKKKs gene family was divided into three different subfamilies. Genomic structures, chromosomal locations, consensus motifs, and promoter were analyzed in all the subfamilies. Gene expression analysis of MAPKKKs under different developmental stages, organs, and stress conditions were carried out to study their roles in *Brachypodium* growth and development. The Diurnal tool was employed and 41 clock-associated MAPKKK genes were identified. Finally, the co-expression network of MPKs, MKKs and MAPKKKs members in *Brachypodium* was constructed using 144 RNA-seq and microarray datasets. Our results will not only provide the foundation for the further study of MAPK cascade signal pathways in *Brachypodium*, but also contribute to better understanding the molecular mechanism of development and stresses signal transduction in *Brachypodium* and beyond.

## Materials and methods

### Databases search and sequences analysis

To identify the MAPKKK genes, the *Brachypodium* protein sequences (*B.distachyon*_192_peptide.fa.bz2) available in PlantGDB (http://www.plantgdb.org/XGDB/phplib/download.php?GDB=Bd) were downloaded to construct a local protein database and local BLASTP search were performed using a total of 171 known MAPKKKs protein sequences (Supplementary Table S1) collected from The *Arabidopsis* information Resource(TAIR), Rice Genome Annotation Project (RGAP), and National Center of Biotechnology Information (NCBI). The identity and *e*-value threshold was set to 50% and 1e-5 (Chambaud et al., 2001), respectively. Further, a self-BLAST of the obtained sequences was carried out to remove the redundancy. Finally, all of the candidates were submitted to the NCBI Batch CD- search (http://www.ncbi.nlm.nih.gov/Structure/bwrpsb/bwrpsb.cgi) and PAFM (http://pfam.sanger.ac.uk/) databases to confirm the presence and integrity of the kinase domain and 86 proteins were remained, which were considered as the BdMAPKKKs. The theoretical isoelectric point (PI) and molecular weight (MW) of these proteins were calculated by the Compute pI/Mw tool in the ExPASy database (http://www.expasy.org/). The subcellular localization predictions were performed using WoLF PSORT tool (http://wolfpsort.org/aboutWoLF_PSORT.html).

### Multiple sequence alignments and phylogenetic tree construction

The full-length predicted MAPKKKs protein sequences in *Brachypodium* and the identified MAPKKKs in *Arabidopsis* and rice were multi-aligned using the MUSCLE program (Edgar, 2004). The phylogenetic tree were constructed basing on the alignment employing neighbor-joining (NJ) and maximum likelihood (ML) method using MEGA 7.0 software (Kumar et al., 2016) with the best model chose by the Akaike information criterion(AIC) implement in ProtTest 2.4 (Abascal et al., 2005). Bootstrap test method was adopted to evaluate the reliability of the tree and the replicate was set to 1000.

### Chromosomal location, gene duplication, and gene structure analysis

The chromosomal location and gene structure information were obtained from PlantGDB database (http://www.plantgdb.org/BdGDB/). And the remaining genes were mapped to chromosomes according to their position using MapInspect program (http://mapinspect.software.informer.com/). The criteria for tandem duplicated genes analysis was used as follows: (1) the alignment length covered >70% of the longer genes; (2) the alignment region had an identity >70%; (3) only one duplication event was counted for tightly linked genes (Gu et al., 2002). The chromosomal segment duplicated MAPKKK genes in *Brachypodium* were identified by searching Plant Genome Duplication Database (PGDD: http://chibba.agtec.uga.edu/duplication/) and the duplicated MAPKKK gene pairs and their corresponding Ka and Ks values for *Brachypodium* with rice, maize, and *Arabidopsis* were also characterized. For the tandem duplicated genes of *Brachypodium*, we aligned the protein sequences of the gene pairs using CLUSTALW2.0 (Larkin et al., 2007) and then used the protein alignments to guide CDS alignments by PAL2NAL (Suyama et al., 2006) to calculate Ka and Ks. The MAPKKK genes sequences with their corresponding exon sequences obtained from PlantGDB were submitted to Gene Structure Display Server (GSDS: http://gsds.cbi.pku.edu.cn/) and to display exon/intron structures.

### Gene promoters and MAPKKK gene expression analysis

To study the regulatory elements of MAPKK kinase genes, 2 kb upstream region sequences of each gene were downloaded from *B. distachyon* genome database (http://mips.helmholtz-muenchen.de/plant/brachypodium/download/index.jsp). All the sequences were submitted to PlantCARE database (http://bioinformatics.psb.ugent.be/webtools/plantcare/html/) to identify the cis-acting regulatory elements. Also in order to gain insight into expression characteristics of each member of the MAPKKKs in different tissues, development stages, and diverse stresses, transcriptome sequencing, and microarray data were obtained from Gene Expression Omnibus (GEO), EBI ArrayExpress database, and PLEXdb. The access numbers and sample information were listed in Supplementary Table S2. The heatmaps and hierarchical clusters were created by MEV v4.9 software (MultiExperiment Viewer, http://www.tm4.org/mev.html). The Diurnal tool constructed by Mockler Lab (http://diurnal.mocklerlab.org/) basing on the expression data was used to identify the clock-associated BdMAPKKK genes with the correlation cutoff value set 0.8.

### Construction and visualization of co-expression network

To confirm interaction relationships among the three main members of MPKs. MKKs, MAPKKKs in the MAPK signal pathway, we first searched the co-expression databases ATTED-II (http://atted.jp/; Obayashi et al., 2009) to get the information of *Arabidopsis*. The IDs of *Arabidopsis* MAPKKs were used as a query to search the databases and the candidate members MAPKKKs and MAPKs in the same cascade were selected with the Rank of MR value set 500. And then, the co-expression networks of *Arabidopsis* were constructed using Cytoscape 2.6.0 (Shannon et al., 2003). For *B. distachyon*, the total of 146 datasets including 9 transcriptome data and 137 microarray data we obtained were used to constructed the co-expression matrix and were calculated MR value (Obayashi and Kinoshita, 2009) for MAPKKKs and MAPKs according to the method adopted in the ATTED-II database construction, and then the MR value lower than 1500 were filter out for constructing co-expression networks.

## Results and discussion

### Genome-wide identification and annotation of the MAPKKK family in *Brachypodium*

Availability of *Brachypodium* complete genome sequences made it possible to identify and annotate all the members of MAPKKK gene family in this model species. Local BLAST search was performed using 171 known MAPKKKs protein sequences as a query to search the *Brachypodium* protein database, which resulted in 96 hits as candidate sequences. These hits were then filtered and only 86 MAPKKKs protein sequences with complete kinase domain remained, which was encoded by 73 MAPKKK genes. As there was no standard nomenclature, the name of predicted MAPKKK genes were designated as *BdMAPKKK1* to *BdMAPKKK73* based on the nomenclature rule used in *Arabidopsis*. The nomenclature of different transcripts encoded by one gene resulting from different pattern of intron splicing share the same gene number with an additional decimal part, such as point 1 or 2 and so on. Previous studies have reported that there were 74 putative MAPKKK genes in maize (Kong et al., 2013), 75 MAPKKKs in rice (Rao et al., 2010), 78 MAPKKKs in cotton (Yin et al., 2013), and 80 MAPKKKs in *Arabidopsis* (Ichimura et al., 2006) as well as 62 MAPKKKs in grape (Çakır and Kılıçkaya, 2015), which indicated the number of MAPKKKs in plant was relatively conserved. The detailed information of BdMAPKKK genes identified in present study, including accession numbers, the length of cDNA and amino acid, molecular weight (Mw), isoelectric point (pI), subcellular localization were listed in Table 1.

**Table 1:** **Characteristics of MAPK kinase kinase (MAPKKKs) in Brachypodium**.

**Family**	**Bd gene name**	**Gene model**	**Chr**	**cDNA**	**Nucleotide features**				**Protein features**						**Subcellular localization prediction**
					**5′UTR**	**CDS**	**3′UTR**	**Intron**	**Length**	**N-ter**	**C-ter**	**Kinase Domain**	**MW (kD)**	**PI**	
MEKK subfamily	*BdMAPKKK1*	Bradi1g07650	1	2883	128	2280	475	10	759	372	128	259	82.71	9.32	Peroxisome
	*BdMAPKKK2*	Bradi1g10970	1	2305	0	1941	364	7	646	375	18	253	69.76	5.19	Chloroplast
	*BdMAPKKK3*	Bradi1g41850	1	1701	0	1701	0	0	566	98	216	252	60.54	6.29	Chloroplast
	*BdMAPKKK4*	Bradi1g58810	1	2293	0	2190	103	10	729	352	118	259	79.39	9.3	Chloroplast
	*BdMAPKKK5*	Bradi1g65500	1	924	0	924	0	0	307	3	48	256	32.29	10.41	Chloroplast
	*BdMAPKKK6*	Bradi1g67397	1	2652	153	1905	594	7	634	354	27	253	68.89	6.23	Nucleus
	*BdMAPKKK7*	Bradi2g17800	2	1557	0	1557	0	2	518	10	257	251	54.35	4.82	Chloroplast
	*BdMAPKKK8*	Bradi2g17820	2	1505	77	1428	0	0	475	10	212	253	50.31	4.52	Chloroplast
	*BdMAPKKK9*	Bradi2g17830	2	1650	0	1650	0	0	549	13	281	255	57.92	4.44	Cytoplasmic
	*BdMAPKKK10*	Bradi2g17840	2	1326	0	1326	0	0	441	8	182	251	46.63	4.74	Chloroplast
	*BdMAPKKK11*	Bradi2g47480	2	1467	0	1467	0	0	488	9	229	250	52.09	4.77	Cytoplasmic
	*BdMAPKKK12*	Bradi2g47490	2	1539	93	1446	0	0	481	9	225	247	51.38	4.58	Chloroplast
	*BdMAPKKK13*	Bradi2g47500	2	1448	65	1383	0	0	460	9	199	252	48.52	4.91	Cytoplasmic
	*BdMAPKKK14*	Bradi2g47510	2	1607	96	1455	56	0	484	9	226	249	52.34	5.15	Mitochondria
	*BdMAPKKK15*	Bradi3g10887	3	1419	0	1419	0	0	472	8	210	254	49.01	5.04	Cytoplasmic
	*BdMAPKKK16.1*	Bradi3g36080.1	3	2464	0	2043	421	16	680	122	298	260	74.83	6.01	Nucleus
	*BdMAPKKK16.2*	Bradi3g36080.2	3	2658	0	1980	678	14	659	122	277	260	72.19	5.61	Nucleus
	*BdMAPKKK17*	Bradi3g45790	3	2110	0	2067	43	10	688	287	145	256	75.15	9.08	Mitochondria
	*BdMAPKKK18*	Bradi3g51380	3	3104	0	2676	428	10	891	408	227	256	96.73	9.62	Nucleus
	*BdMAPKKK19*	Bradi3g57740	4	2143	0	1587	556	7	528	270	6	252	57.82	6.96	Chloroplast
	*BdMAPKKK20*	Bradi4g22760	4	1992	0	1992	0	10	663	252	155	256	71.89	9.53	Nucleus
	*BdMAPKKK21*	Bradi4g29500	4	2599	0	2055	544	16	684	104	318	262	74.63	6.06	Nucleus
	*BdMAPKKK22.1*	Bradi5g10670.1	5	2348	0	2091	257	10	696	291	149	256	75.56	9.19	Nucleus
	*BdMAPKKK22.2*	Bradi5g10670.2	5	2345	0	2088	257	10	695	291	148	256	75.48	9.19	Nucleus
	*BdMAPKKK23*	Bradi5g18180	5	2691	0	2691	0	10	896	410	229	257	97.56	9.7	Nucleus
	*BdMAPKKK24.1*	Bradi5g24870.1	5	4415	0	4038	377	23	1345	18	1073	254	148.9	5.93	Nucleus
	*BdMAPKKK24.2*	Bradi5g24870.2	5	4322	0	3945	377	24	1314	18	1042	254	146	5.9	Cytoplasmic
	*BdMAPKKK24.3*	Bradi5g24870.3	5	4169	0	3792	377	23	1263	18	991	254	140.9	5.87	Cytoplasmic
Raf subfamily	*BdMAPKKK25.1*	Bradi1g04080.1	1	1770	292	1152	326	5	383	58	64	261	42.45	8.11	Cytoplasmic
	*BdMAPKKK25.2*	Bradi1g04080.2	1	1773	291	867	615	5	287	59	1	227	31.93	8.91	Cytoplasmic
	*BdMAPKKK26*	Bradi1g14000	1	1993	463	1131	399	5	376	71	29	276	41.43	8.52	Cytoplasmic
	*BdMAPKKK27*	Bradi1g14010	1	1131	0	1131	0	5	376	71	29	276	41.45	8.18	Cytoplasmic
	*BdMAPKKK28*	Bradi1g20390	1	1329	0	1329	0	10	442	165	19	258	49.11	7.15	Cytoplasmic
	*BdMAPKKK29*	Bradi1g28110	1	1593	0	1593	0	14	530	257	25	248	59.05	6.31	Chloroplast
	*BdMAPKKK30.1*	Bradi1g28950.1	1	3923	0	3660	263	7	1219	942	15	262	134.2	5.48	Nucleus
	*BdMAPKKK30.2*	Bradi1g28950.2	1	3692	0	3660	32	7	1219	942	15	262	134.2	5.48	Nucleus
	*BdMAPKKK31*	Bradi1g30720	1	3938	0	3321	617	8	1106	833	10	263	118.7	5.53	Nucleus
	*BdMAPKKK32*	Bradi1g35350	1	1229	0	1215	14	1	404	106	44	254	45.24	9.26	Chloroplast
	*BdMAPKKK33.1*	Bradi1g45040.1	1	3822	0	3222	600	12	1073	799	22	252	117.6	5.51	Chloroplast
	*BdMAPKKK33.2*	Bradi1g45040.2	1	3909	0	3129	780	11	1042	799	2	241	114	5.39	Chloroplast
	*BdMAPKKK34*	Bradi1g47570	1	4540	0	4002	538	8	1333	1056	14	263	142.2	5.75	Nucleus
	*BdMAPKKK35*	Bradi1g74480	1	3521	0	3006	515	12	1001	719	30	252	110	6.23	Chloroplast
	*BdMAPKKK36*	Bradi2g00670	2	2080	642	1113	325	2	370	66	50	254	41.07	9.19	Nucleus
	*BdMAPKKK37*	Bradi2g06260	2	2635	477	1686	472	2	561	265	40	256	63.4	9.36	Nucleus
	*BdMAPKKK38*	Bradi2g15560	2	1414	0	1140	274	5	379	75	30	274	42.12	8.01	Nucleus
	*BdMAPKKK39*	Bradi2g19590	2	2466	441	1788	237	2	595	284	55	256	66.38	8.5	Nucleus
	*BdMAPKKK40*	Bradi2g44910	2	1477	0	1164	313	5	387	83	30	274	42.92	7.69	Chloroplast
	*BdMAPKKK41*	Bradi2g46340	2	2654	0	2409	245	15	802	546	8	248	89.18	5.91	Nucleus
	*BdMAPKKK42*	Bradi2g49700	2	2671	513	1785	373	2	594	283	55	256	66.33	8.94	Nucleus
	*BdMAPKKK43*	Bradi2g49790	2	1574	0	1353	221	11	450	146	48	256	50.69	6.05	Cytoskeleton
	*BdMAPKKK44*	Bradi2g57470	2	1482	0	1482	0	10	493	212	21	260	55.24	9.01	Chloroplast
	*BdMAPKKK45*	Bradi3g01850	3	2010	0	1803	207	13	600	323	29	248	66.84	6.9	Cytoplasmic
	*BdMAPKKK46*	Bradi3g05520	3	1821	247	1272	302	1	423	142	27	254	46.99	7.32	Cytoplasmic
	*BdMAPKKK47.1*	Bradi3g08260.1	3	3209	0	2562	647	11	853	589	12	252	93.22	5.45	Chloroplast
	*BdMAPKKK47.2*	Bradi3g08260.2	3	3316	0	2481	835	10	826	589	0	237	90.17	5.4	Chloroplast
	*BdMAPKKK48.1*	Bradi3g09170.1	3	2559	0	2379	180	15	792	536	8	248	88.34	6.25	Nucleus
	*BdMAPKKK48.2*	Bradi3g09170.2	3	2508	0	2352	156	15	783	536	6	240	87.35	6.74	Nucleus
	*BdMAPKKK49*	Bradi3g13050	3	1527	0	1527	0	12	508	261	44	203	57.59	5.12	Nucleus
	*BdMAPKKK50*	Bradi3g13060	3	1937	0	1665	272	13	554	285	29	240	62.57	6.43	Nucleus
	*BdMAPKKK51*	Bradi3g18150	3	1870	193	1254	423	1	417	136	27	254	45.99	8.41	Cytoplasmic
	*BdMAPKKK52.1*	Bradi3g27120.1	3	3316	0	2907	409	12	968	689	27	252	107.1	5.84	Nucleus
	*BdMAPKKK52.2*	Bradi3g27120.2	3	3313	0	2904	409	12	967	689	27	251	107	5.84	Nucleus
	*BdMAPKKK53*	Bradi3g44707	3	2565	0	2292	273	16	763	493	16	254	83.15	7.95	Chloroplast
	*BdMAPKKK54*	Bradi3g45660	3	2196	0	2196	0	12	731	448	31	252	81.79	6.89	Nucleus
	*BdMAPKKK55*	Bradi3g47600	3	1550	0	1053	497	5	350	26	64	260	39.42	6.97	endoplasmic reticulum
	*BdMAPKKK56*	Bradi3g48360	3	1708	0	1452	256	11	483	202	27	254	53.93	8.32	Chloroplast
	*BdMAPKKK57*	Bradi3g59510	3	3683	0	3345	338	12	1114	840	22	252	122.7	5.2	Nucleus
	*BdMAPKKK58*	Bradi3g60210	3	3919	0	3348	571	7	1115	842	10	263	122.7	5.48	Nucleus
	*BdMAPKKK59*	Bradi4g02367	4	1277	0	1149	128	5	382	74	30	278	41.88	5.36	Chloroplast
	*BdMAPKKK60*	Bradi4g02900	4	3653	0	3231	422	7	1076	796	17	263	118.5	5.21	Nucleus
	*BdMAPKKK61*	Bradi4g04470	4	2788	0	2277	511	12	758	478	28	252	83.84	7.35	Nucleus
	*BdMAPKKK62*	Bradi4g09990	4	2701	0	2226	475	12	741	463	26	252	82.38	7.66	Nucleus
	*BdMAPKKK63.1*	Bradi4g36880.1	4	2227	116	1779	332	15	592	311	32	249	65.66	5.7	Cytoplasmic
	*BdMAPKKK63.2*	Bradi4g36880.2	4	2296	116	1743	437	14	580	311	20	249	64.48	5.48	Cytoplasmic
	*BdMAPKKK63.3*	Bradi4g36880.3	4	2511	116	1581	814	13	526	311	2	213	58.22	5.58	Cytoplasmic
	*BdMAPKKK64*	Bradi4g38400	4	2934	0	2304	630	15	767	496	17	254	83.66	6.48	Chloroplast
	*BdMAPKKK65.1*	Bradi4g41870.1	4	2062	178	1617	267	14	538	264	26	248	58.75	4.91	Cytoplasmic
	*BdMAPKKK65.2*	Bradi4g41870.2	4	1551	60	1224	267	12	407	133	26	248	45.43	4.99	Nucleus
	*BdMAPKKK66*	Bradi5g21330	5	3121	0	2289	832	14	762	496	12	254	83.42	6.96	Chloroplast
	*BdMAPKKK67*	Bradi5g26917	5	1689	0	1689	0	14	562	285	29	248	62.76	5.55	Cytoplasmic
ZIK subfamily	*BdMAPKKK68*	Bradi1g23320	1	1204	0	873	331	1	290	20	12	258	32.71	5.96	Cytoplasmic
	*BdMAPKKK69*	Bradi1g23970	1	2678	122	2043	513	6	680	25	399	256	76.88	5.51	Nucleus
	*BdMAPKKK70*	Bradi2g39347	2	2381	0	1932	449	8	643	25	355	263	72.45	4.88	Nucleus
	*BdMAPKKK71*	Bradi3g51457	3	2106	0	1848	258	6	615	25	333	257	68.33	5.02	Chloroplast
	*BdMAPKKK72*	Bradi4g41940	4	1707	136	1101	470	4	366	2	145	219	41.14	6.44	Cytoplasmic
	*BdMAPKKK73*	Bradi4g44427	4	2100	0	1938	162	3	645	32	340	273	70.07	5.8	Nucleus

*Chr, chromosome; UTR, Untranslated regions; CDS, Coding DNA sequence; N-ter, N terninal; C-ter, C terninal; MW, Molecular weight; IP, Isoelectric ponit*.

Furthermore, the length of cDNA of BdMAPKKK gene ranged from 924 bp (*BdMAPKKK5*) to 4540 bp (*BdMAPKKK35*) and their protein sequences constituted 287–1845 amino acids (aa). The Mw of these proteins ranged from 31.93 kDa (*BdMAPKKK26.2*) to 148.94 kDa (*BdMAPKKK24.1*) and the pI value ranged from 4.44 (*BdMAPKKK9*) to 10.41 (*BdMAPKKK5*). The subcellular localization indicated that most of the predicted MAPKKK proteins localized in nuclear (41.86%), followed by chloroplast (27.91%), and cytoplasm (24.42%). Additionally, *BdMAPKKK1, BdMAPKKK43*, and *BdMAPKKK55* were predicted to localize in peroxisome, cytoskeleton, and endoplasmic reticulum, respectively.

### Phylogenetic and gene duplication analysis of BdMAPKKKs

**Figure 1:**
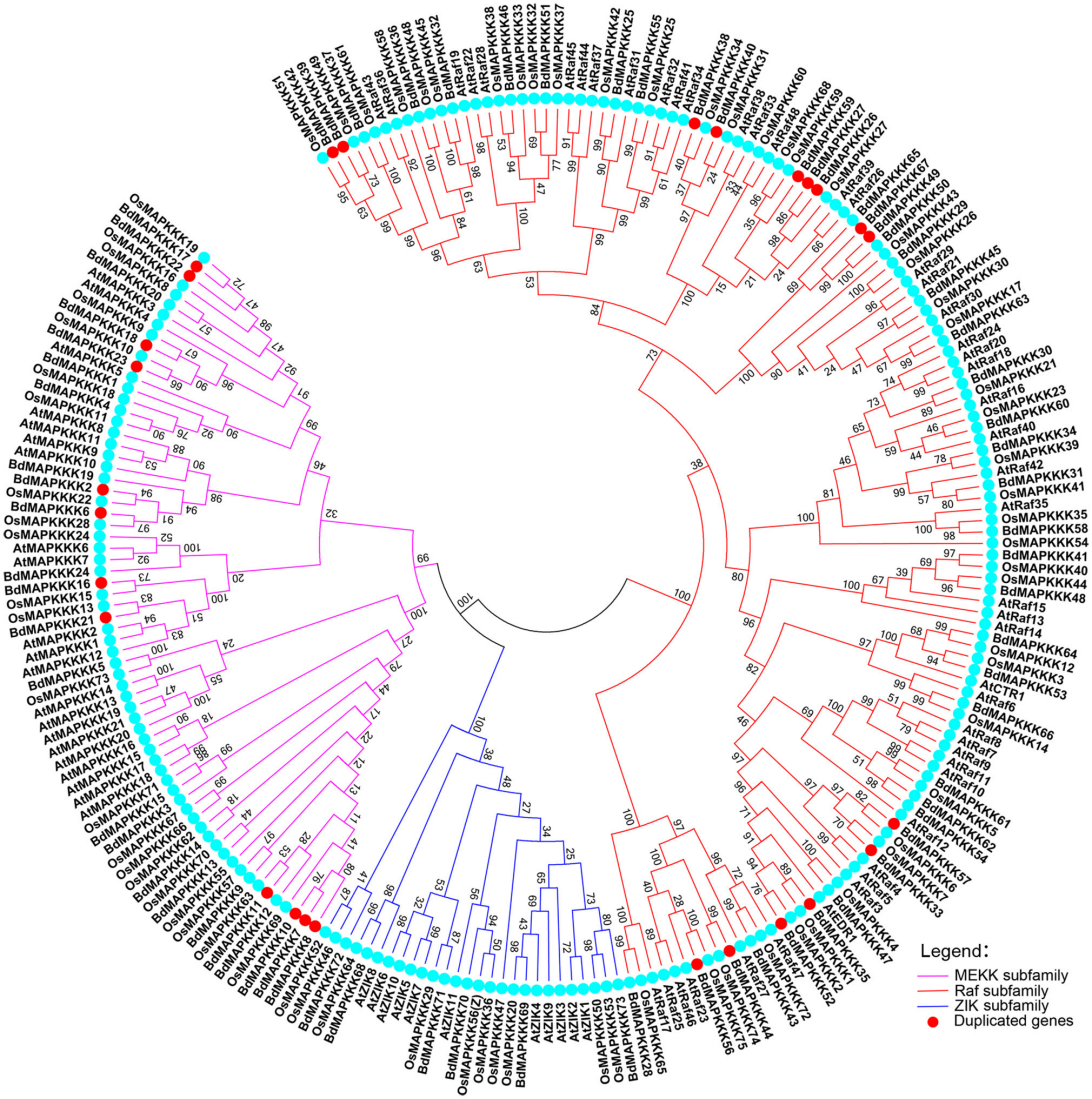
**Phylogenetic tree of MAPKKKs from *Brachypodium*, rice, and *Arabidopsis***. Neighbor-joining tree was created using MEGA7.0 program with the best model JTT+G (G set 0.9), using full length sequences of 73 *Brachypodium*, 75 rice, and 80 *Arabidopsis* MAPKKK proteins. The bootstrap value was set 1000 replicates.

To characterize the phylogenetic relationships of MAPKKKs in *Brachypodium* with that of *Arabidopsis* and rice, the phylogenetic trees were constructed by employing NJ method and ML method, respectively (Figure 1). The topological structures of these two trees were almost similar except for a few clades. The three MAPKKK subfamilies MEKK, Raf, and ZIK of *Arabidopsis* and rice could be clustered together, respectively, implying the constructed phylogenetic tree was reliable. On the basis of phylogenetic analysis, the BdMAPKKK family could also be classified into three subfamilies. There were 28 BdMAPKKK proteins encoded by 24 BdMAPKKK genes belonging to MEKK subfamily, 6 BdMAPKKK proteins encoded by 6 genes to ZIK subfamily and 52 BdMAPKKKs encoded by 43 genes grouped into Raf subfamily which was the largest subfamily of MAPKKKs. By comparison with rice and maize, the number of each subfamily of MAPKKK showed no significantly variations, but it was significantly less than that of soybean due to the higher duplication ratio of soybean genome (Schmutz et al., 2010; Table 2).

**Table 2:** **The number of MAPKKK gene family in Arabidopisis, rice, maize, cotton, soybean, and Brachypodium**.

**Species**	**MEKK**	**Raf**	**ZIK**	**Total**
*Arabidopsis*	21	48	11	80
Rice	22	43	10	75
Maize	22	46	6	74
Cotton	22	44	12	78
Soybean	34	92	24	150
Grape^*^	21	29	12	62
*Brachypodium*	24	43	6	73

* *Data obtained from Çakır and Kılıçkaya (2015)*.

**Figure 2:**
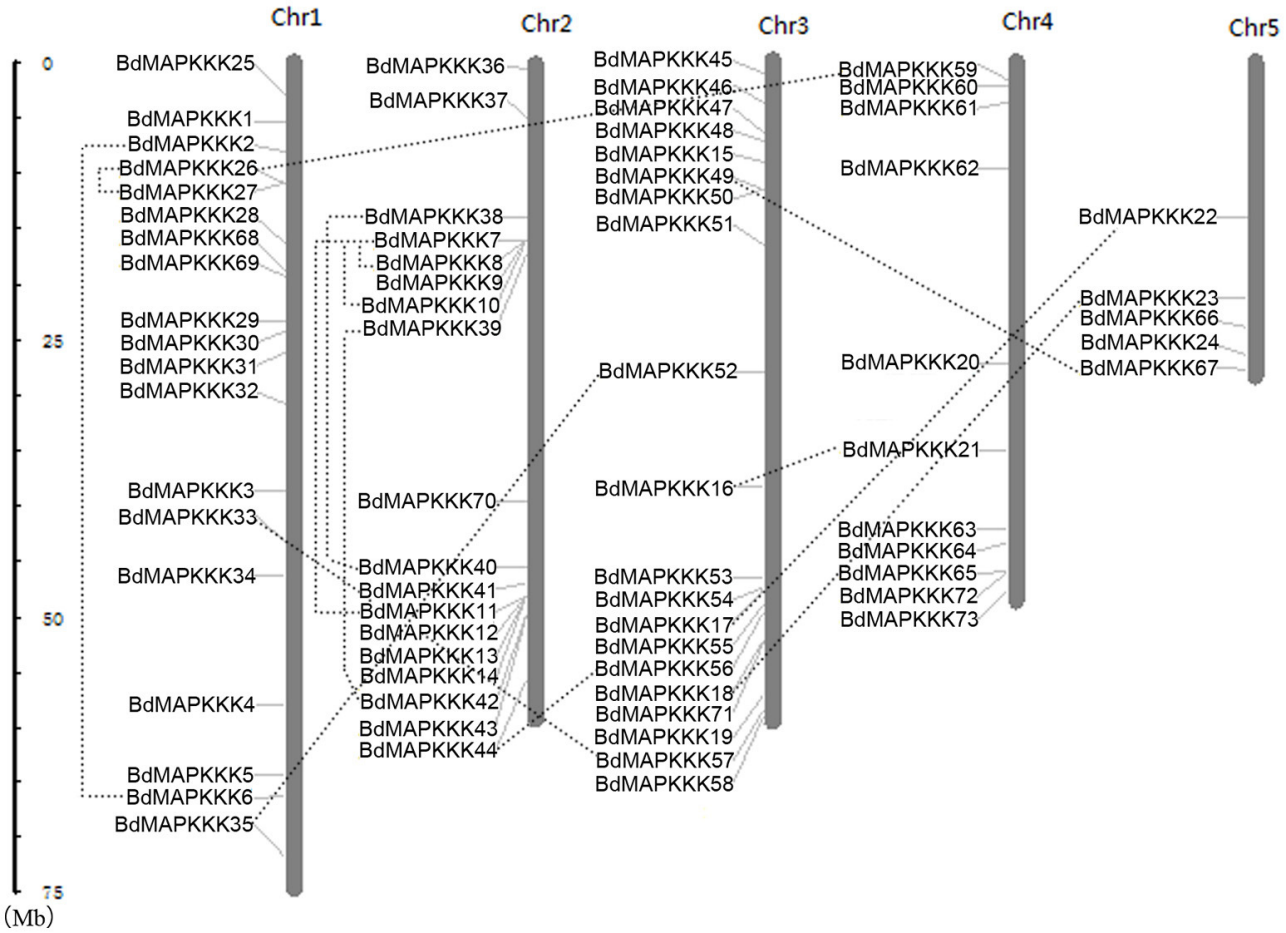
**Chromosomal distributions of MAPKKK genes in *Brachypodium* genome**. The duplicated MAPKKK gene pairs were connected by dotted line. Chr: chromosome.

Furthermore, chromosomal locations of all the 73 BdMAPKKK members were investigated and they were dispersed on all of the five chromosomes (Figure 2). Among them, 19 were mapped on chromosome 1, 18 on chromosome 2 and 20 on chromosome 3 as well as 11 on chromosome 4 and only 5 on chromosome 5. The gene duplication analysis showed that only three clusters (*BdMAPKKK7*-*BdMAPKKK8, BdMAPKKK7*-*BdMAPKKK10*, and *BdMAPKKK26*-*BdMAPKKK27*) were generated by tandem duplication (Table 3). Phylogenetic analysis suggested that the three pairs were also clustered together, respectively, and each cluster only contained *Brachypodium* MAPKKK genes, which indicated the duplications event had happened after the rice and *Brachypodium* diverged. And then, the divergent time between the tandem genes were calculated, showing the duplication happened 15.5–53.5 Myr later than the time of the *Brachypodium* and rice divergence (International Brachypodium Initiative, 2010), which further supported the evolutionary relationships obtained from phylogenetic analysis. In addition, 12 pairs of BdMAPKKK genes were identified by chromosomal segment duplication analysis (Table 3), of which 4 pairs (*BdMAPKKK2*-*BdMAPKKK6, BdMAPKKK7*-*BdMAPKKK11, BdMAPKKK38*-*BdMAPKKK40*, and *BdMAPKKK39*-*BdMAPKKK42*) located on chromosome 1 and 2 were originated from recent inter-chromosomal gene duplication events. According to the phylogenetic tree, we found two members of the chromosomal segment duplication genes pairs except for *BdMAPKKK49*-*BdMAPKKK67* were first clustered together with one OsMAPKKK genes, and then clustered together into one clade, which indicated the chromosomal segment duplication of BdMAPKKK gene may happen before the divergence of the rice and *Brachypodium* but after the divergence of monocots and eudicots. And the divergent time of all chromosomal segment duplicated gene pairs in *Brachypodium* suggested that the duplication event happened 55.4–128.3 Myr ago and earlier than the time of the *Brachypodium* and rice divergence happening 40.6–53.9 Myr ago, which strongly supported the phylogenetic analysis mentioned above and also indicated that the tandem duplication event was younger than chromosomal segment duplication. However, the pair of *BdMAPKKK49*-*BdMAPKKK67* genes was first cluster together with *BdMAPKKK50* without the participation of OsMAPKKK genes, possibly resulting from the higher similarity (protein similarity 61.23%) of *BdMAPKKK50* with *BdMAPKKK49* (that of *BdMAPKKK67* with *BdMAPKKK 49* was 51.04%) and the orthologous genes may lost in rice during evolution. As for the number of duplicated BdMAPKKK genes, we can concluded that the duplication events concentrated on MEKK and Raf subfamily, while no happened in ZIK subfamily, which consistent with the least members of ZIK subfamily. To conclude, gene duplication, especially chromosomal segment duplication acted vital roles in MAPKKK genes expansion in *B. distachyon*.

**Table 3:** **The duplicated BdMAPKKK genes, Ka/Ks analysis, and divergent time calculation**.

**Locus1**	**Chr**	**Locus2**	**Chr**	**Identity (%)**	**Ka**	**Ks**	**Ka/Ks**	**Divergence time (million years)**	**Duplication Type**	**PCC**
BdMAPKKK21	4	BdMAPKKK16	3	65.53	0.1927	1.2025	0.160249	98.57	CSD	0.94
BdMAPKKK22	5	BdMAPKKK17	3	61.15	0.2864	0.8544	0.335206	70.03	CSD	0.61
BdMAPKKK23	5	BdMAPKKK18	3	73.52	0.1511	0.7296	0.2071	59.80	CSD	0.74
BdMAPKKK2	1	BdMAPKKK6	1	68.34	0.3325	1.9176	0.173394	157.18	CSD	–
BdMAPKKK26	1	BdMAPKKK59	4	67.59	0.2313	1.4031	0.164849	115.01	CSD	–
BdMAPKKK57	3	BdMAPKKK33	1	67.82	0.1978	0.8084	0.244681	66.26	CSD	0.75
BdMAPKKK52	3	BdMAPKKK35	1	61.84	0.267	0.8802	0.30334	72.15	CSD	0.76
BdMAPKKK38	2	BdMAPKKK40	2	83.06	0.0954	0.676	0.141124	55.41	CSD	0.40
BdMAPKKK39	2	BdMAPKKK42	2	56.54	0.2911	1.3928	0.209003	114.16	CSD	0.45
BdMAPKKK56	3	BdMAPKKK44	2	59.33	0.3391	1.519	0.223239	124.51	CSD	0.31
BdMAPKKK49	3	BdMAPKKK67	5	51.04	0.4184	1.1184	0.374106	91.67	CSD	–
BdMAPKKK11	2	BdMAPKKK7	2	67.05	0.4393	1.0533	0.41707	86.34	CSD	0.09
BdMAPKKK8	2	BdMAPKKK7	2	73.56	0.1977	0.6532	0.302664	53.54	TD	0.51
BdMAPKKK10	2	BdMAPKKK7	2	88.19	0.1085	0.3825	0.28366	31.35	TD	0.56
BdMAPKKK26	1	BdMAPKKK27	1	98.14	0.0091	0.1896	0.047996	15.54	TD	0.81

*Chr, chromosome; Identity, proetein sequences similarity; CSD, chromosomal segment duplication; TD, tandem duplication; PCC, Pearson's correlation coefficients*.

To analyze the selective pressure acting during the evolution of MAPKKK genes, we further identified homology of the BdMAPKKK genes with rice, maize and *Arabidopsis* and also calculated the Ka/Ks ratio for each pairs (Supplementary Table S3). The Ka/Ks values were significantly < 1 in all pairs, providing a crude indication that the strong purifying selection played an important roles in the constraint on the MAPKKK genes functions, which was consistent with the conservation of the MAPKKK genes. In addition, we calculated the protein sequences identity of the chromosomal segment duplicated gene pairs and showed the value was ranged from 51.04% (*BdMAPKKK49*-*BdMAPKKK67*) to 68.34% (*BdMAPKKK2*-*BdMAPKKK6*) except for *BdMAPKKK23*-*BdMAPKKK18* (73.52%) and *BdMAPKKK38*-*BdMAPKKK40* (83.06%) higher than 70% (Table 3), implying the protein sequences have been differentiated a lot during MAPKKK genes evolution. Furthermore, the PCC (Pearson's correlation coefficients) value for each pair of duplicated genes expression were calculated (Table 3). Results showed that the PCC value of two pairs including *BdMAPKKK16*-*BdMAPKKK21* and *BdMAPKKK26*-*BdMAPKKK27* were more than 0.8, and 8 pairs varied from 0.4 to 0.8, while that of the remaining two pairs (*BdMAPKKK56*-*BdMAPKKK44, BdMAPKKK11*-*BdMAPKKK7*) was 0.31 and 0.09, respectively, which roughly indicated the duplicated genes of two pairs have experienced functional divergence. The PCC for the pairs of duplicated genes identity and their expression was 0.19, suggesting there was weak correlation between them.

### Domain and motif analysis of BdMAPKKKs

**Figure 3:**
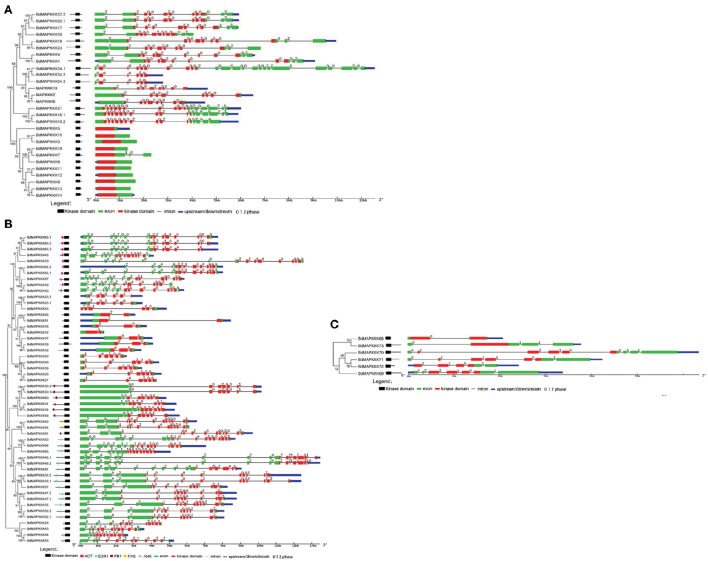
**The intron and exon structure of three MAPKKK subfamilies in *Brachypodium***. Phylogenetic trees were created using MEGA program with the best model JTT+G (G set 0.9). The bootstrap value was set 1000 replicates. The kinase domain relative position was drawn manually and its accurate position was shown in the gene structure picture using red boxes. **(A)** MEKK subfamily. **(B)** Raf subfamily. The additional domains were shown in the draft picture of the domain distribution. **(C)** ZIK subfamily.

**Figure 4:**
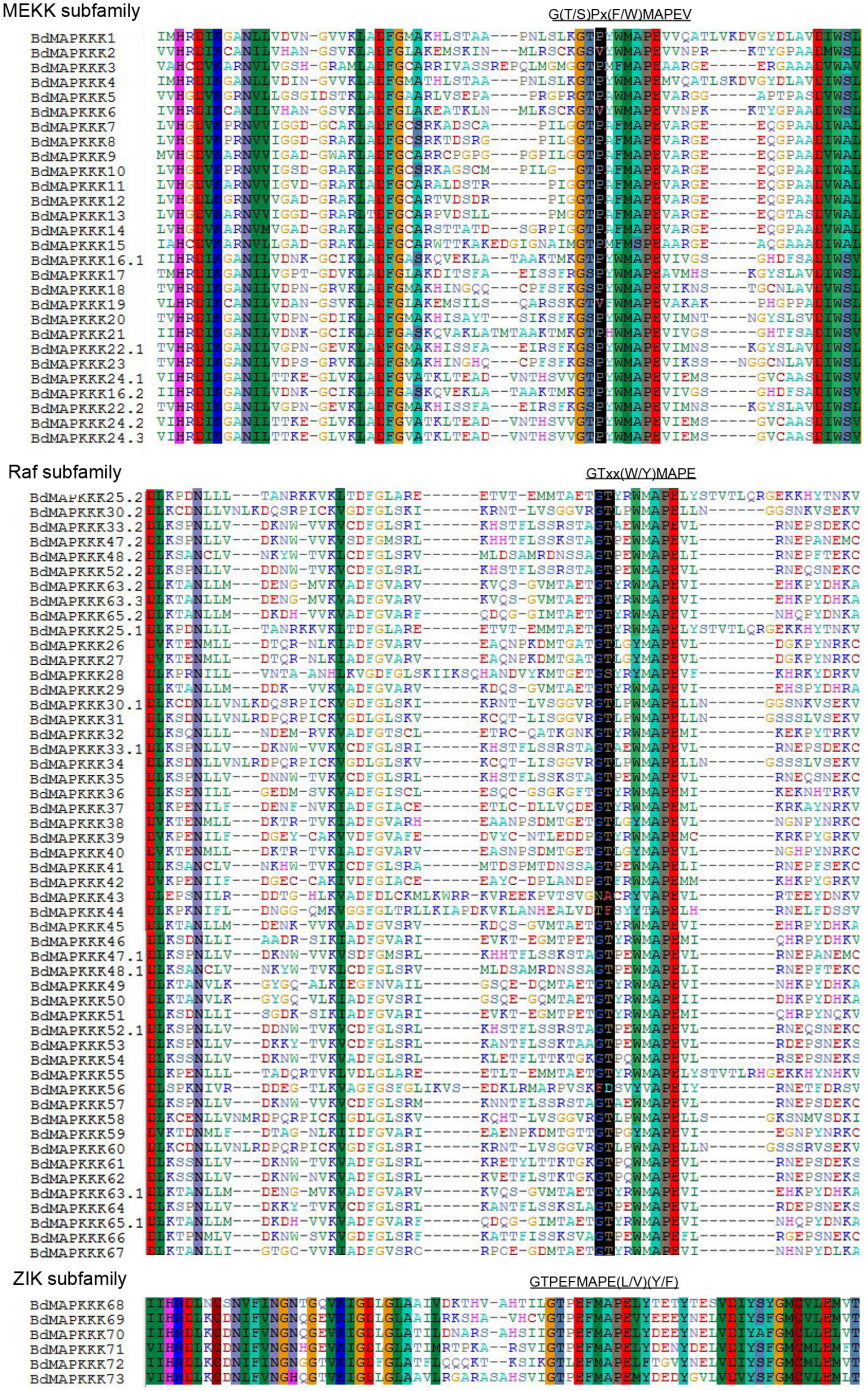
**The alignment of MAPKKK protein kinase domain sequences in *Brachypodium***. Alignment was performed using MUSCLE program. The highlighted part shows the conserved signature motif.

The domains in BdMAPKKKs were analyzed using the NCBI batch CD–Search and PFAM database, and the name and position information were confirmed and roughly drawn in Figure 3. The relative positions of kinase domains in three subfamilies of BdMAPKKKs were found to have various patterns. In MEKK subfamily, the kinase domains were located either at C or N-terminal or central part of the protein sequence (Figure 3A). As for Raf subfamily, there existed a C-terminal kinase domain and a long N-terminal regulatory domain (Figure 3B) whereas all the ZIK members had a long N-terminal kinase domain (Figure 3C). These results were consistent with *Arabidopsis*, rice, maize, and cotton (Ichimura et al., 2006; Rao et al., 2010; Kong et al., 2013; Yin et al., 2013). In addition, the three subfamilies protein sequences of the kinase domain were then extracted to perform multiple alignments using MUSCLE program to detect conserve motif. It was found all the members of ZIK subfamily shared a conserved motif: GTPEFMAPE(L/V)(Y/F) and MEKK group shared G(T/S)Px(F/W)MAPEV motif, as well as Raf shared GTxx(W/Y)MAPE motif (Figure 4). Cautiously, the conserve signature of Raf subfamily was also found in receptor-like protein kinase of plant serine/threonine kinase protein family in *Brachypodium*, so the identification of the Raf subfamily should not just depend on the consensus motif. Simultaneously, a self blast using the separated kinase domain protein sequences was performed and the result suggested the similarity of most of BdMAPKKKs were lower than 50% and showed that the kinase domains may existed remarkable variation in different members, implying they played important roles in the function differentiation of BdMAPKKKs.

Furthermore, there also existed other domains in Raf subfamily except for kinase domain (Figure 3B), including the aspartokinase chorismate mutase and TyrA (ACT) domain, EDR1 domain, Phox and Bem1p (PB1) domain, PAS domain, and ankyrin repeats (ANK) domain, where they almost located in the long N-terminal of BdMAPKKKs. These domains also existed in *Arabidopsis* and grape MAPKKKs (Ichimura et al., 2006; Wang et al., 2014), which may play important roles in the regulation pathway of BdMAPKKKs acting in, specifically in signal transduction.

### Gene structure analysis of BdMAPKKK genes

**Figure 5:**
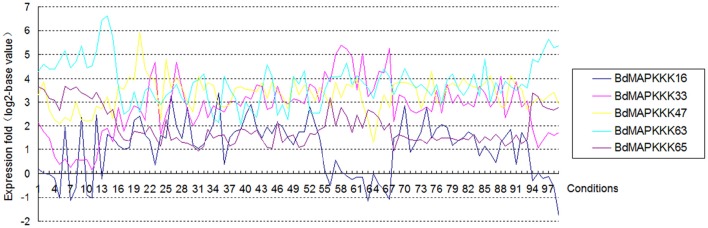
**The expression of each splicing transcripts of the five detected BdMAPKKK genes using the microarray data**. The ordinate axis represents the fold change of average expression for the transcripts name with point 1 compared with the point 2 in each condition, and the 1 to 99 in abscissa represent the different conditions in E-MEXP-3729, GSE38247, BD3, BD1, and E-MEXP-3918, respectively.

Gene structure analysis could contribute to better understanding its functions, regulation, and evolution (Liu et al., 2015). In order to get some insight into the gene structure of BdMAPKKK genes, the exon/intron organization of them was analyzed (Figure 3). There were 9 (12.33%) members of BdMAPKKK genes having no intron, and interestingly all of them belonged to MEKK subfamily. The remaining BdMAPKKK genes had intron(s) and the number of intron(s) ranged from 1 (*BdMAPKKK5, BdMAPKKK33*, and *BdMAPKKK69*) to 24 (*MAPKKK24.2*), indicating a great variation of intron number presented among BdMAPKKK genes. In MEKK subfamily, the intron numbers showed a greater degree of diversity ranging from 0 to 24, while most of them had 7–16 introns, which was consistent with MEKK subfamily in maize (Kong et al., 2013). In Raf members, there were 1–16 introns present in them. In addition, the 6 ZIK members possessed 1–8 introns, showing more introns than that of maize and rice ZIK subfamily (Rao et al., 2010; Kong et al., 2013). Furthermore, the phases of the splicing sites could be found were various in BdMAPKKK family (Figure 3). However, the numbers, phases and arrangement of introns in the region of the kinase domain are highly conserved within each subfamily (Figure 3, Supplementary Table S4). In MEKK subfamily kinase domains, there were only four patterns of the intron number 0, 7, 8, 10, and two types of intron phase: 0, 2, of which 0, 7 introns (78.57%) and phase 0 are the major pattern of BdMAKKK genes followed. And all the members from Raf kinase domains possessed 1–10 introns, and most of introns splicing sites of these genes tend to adopt phase 0 and few adopted phase 1. The remaining ZIK family genes had 1–5 introns in kinase domains, whereas *BdMAPKKK69* and *BdMAPKKK74* genes had only one intron. And the three intron phases adopted in ZIK subfamily were almost equal. Our results indicated that the intron patterns within subfamily in kinase domains were highly conserved during the evolution of the *Brachypodium* genome, and the close correlation between the phylogeny and exon/intron structure provided an independent criterion for testing the reliability of phylogenetic analysis. In addition, different splicing transcripts were also studied. Most of BdMAPKKK genes had alternative splicing, but only few BdMAPKKK genes splicing transcripts remained kinase domain. The structure analysis of the different spliceosomes indicated that the difference mostly happened in the 3′ terminal of gene resulting from the additional one or two intron (s) insert, except for *BdMAPKKK25* and *BdMAPKKK48* genes produced by the last exon and intron extension, while *BdMAPKKK65* gene was disrupted by another two introns insert in the 5′ terminal. Furthermore, the different splicing position of the majority of BdMAPKKK genes splicing transcripts was located in the outside of the kinase domain, but those of *BdMAPKKK33, BdMAPKKK47*, and *BdMAPKKK63* gene were found to be in the kinase domain. In order to explore which spliceosomes were adopted by BdMAPKKK genes, the expression of each splicing transcripts of five detected BdMAPKKK genes (*BdMAPKKK16, BdMAPKKK33, BdMAPKKK47, BdMAPKKK63, BdMAPKKK65*) were analyzed (Figure 5). All the expression values were obtained from transcript microarray experiments. According to the results, we found that four gene showed significantly difference in the expression of splicing patterns and the transcripts named with a point 1, which could translate the longest size of protein among the various splicing transcripts, showed the higher expression in most of conditions (log_2_-base value of fold change more than 1), implying that they were the predominantly splicing pattern. However, the remaining one gene *BdMAPKKK16* expression showed different feature (Figure 5). The expression of BdMAPKKK16.2 was higher than BdMAPKKK16.1 in PMV and SPMV infection, expansion, and mature zone of leaf in different drought treatment, leaf, heat and Circadian_LLHH LDHH (LLHH: Light day, Light night, Hot day, Hot night; LDHH: Light day, Dark night, Hot day, Hot night) conditions.

### Expression analysis of BdMAPKKK gene at different developmental stages and organs

**Figure 6:**
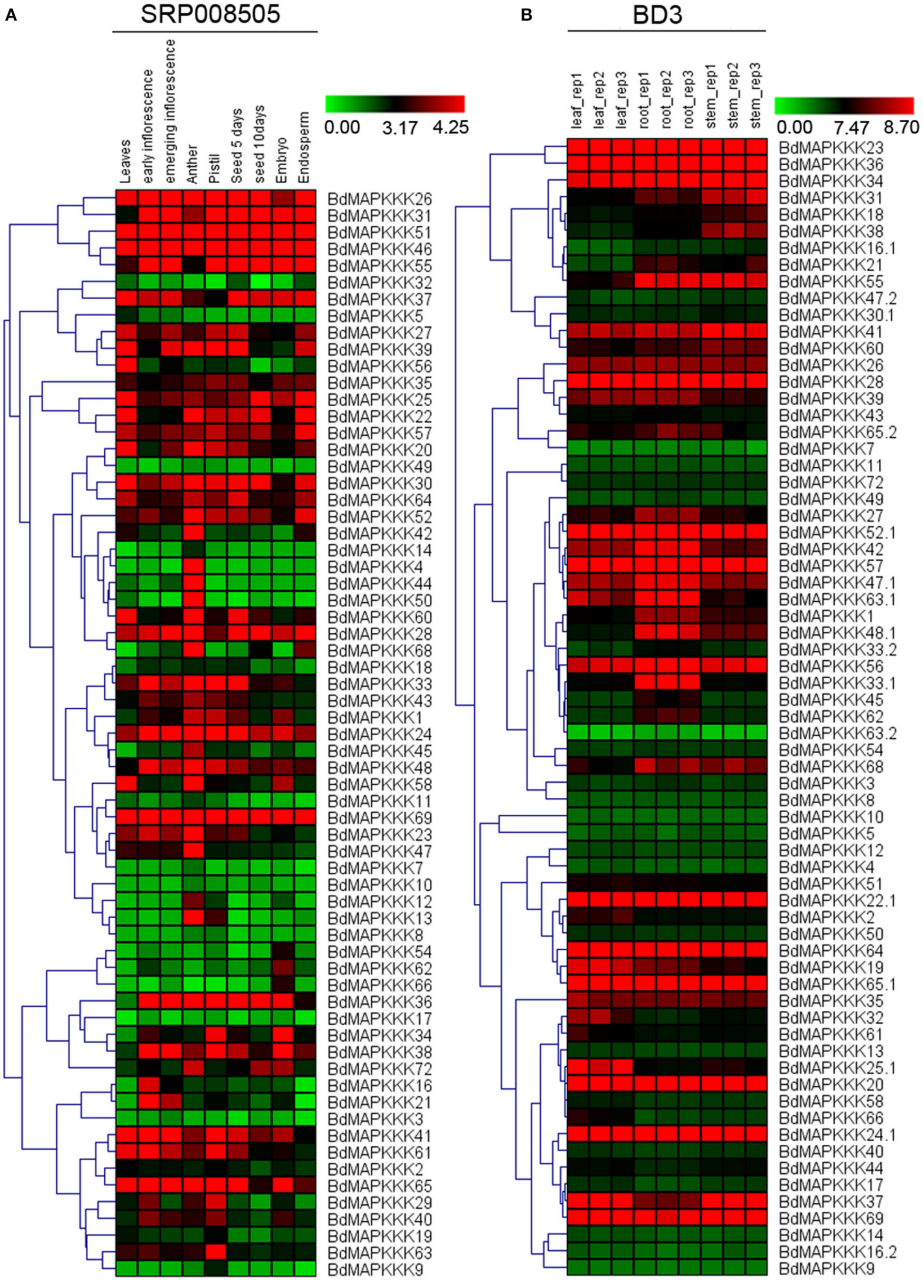
**The expression profile of BdMAPKKK genes in different tissues and organs**. The red and green shading represents the relative high or low expression levels, respectively. **(A)** The expression pattern of BdMAPKKK in different development stages and organs. The color bar represents the original expression value plus1 and then make a log_2_. **(B)** The expression pattern of BdMAPKKK in leaf, root and stem. The color bar represents log_2_ expression value.

To understand the temporal and spatial expression patterns of MAPKKKs in *Brachypodium*, we compared their expression profiles in different organs and developmental tissues, including roots, stems, leaves, seed, anther, pistil, inflorescence, embryo, and endosperm. In this study, microarray datasets of *Brachypodium* were used to investigate the expression patterns of roots, stems, and leaves, respectively, and 68 MAPKKK proteins encoded by 63 MAPKKK genes (22 MEKK subfamily members, 38 Raf subfamily members, and 3 ZIK subfamily members) were detected. For different developmental stage tissues, we employed the RNAseq data (SRP008505) to study BdMAPKKK genes expression patterns and 65 BdMAPKKK genes (22 MEKK subfamily members, 40 Raf subfamily members and 3 ZIK subfamily members) were detected. And then, two visualized hierarchy cluster of different developmental stages and organs were constructed and shown in Figure 6.

In order to identify the differentially expressed genes in specific stages, we calculated the coefficient of variation (CV value) of each BdMAPKKK gene expression (Supplementary Table S5). From the results, we found there was a high fluctuation of the CV value ranging from 14.86 to 259.87% in the nine stages. When we set the CV value lower than 20%, the *BdMAPKKK35* and *BdMAPKKK57* belonged to Raf subfamily were found, which suggest the expressions of two members are stable and may play basal part in all kinds of developmental stages. Also we found that there are 6 genes with a CV value more than 200% including three MEKK subfamily members (*BdMAPKKK4, BdMAPKKK9, BdMAPKKK14*) and three Raf subfamily members (*BdMAPKKK44, BdMAPKKK50, BdMAPKKK66*). According to the Figure 6A, *BdMAPKKK4, BdMAPKKK44, BdMAPKKK50* genes were mostly just expressed in anther, whereas *BdMAPKKK9* and *BdMAPKKK14* were mainly expressed in pistil and anther, suggesting they may be involved in the regulation of reproductive organs development. *BdMAPKKK66* gene almost just expressed in embryo, implying that it may took participated in the reservation of carbohydrate substance. In addition, we found only three genes had the CV value between 150 and 200% and the expression of *BdMAPKKK5* gene (CV value 150.79%) was mostly in leaves, *BdMAPKKK13* gene (CV value 182.05%) almost just expressed in pistil and anther, and *BdMAPKKK60* gene (CV value 192.82%) had a higher expression in anther than other stages, implying the three genes may play special roles in corresponding development stages or organs. Furthermore, we used the fold change method (log_2_-base ratio) with more than 1-fold as the criterion to find differentially expressed genes in the different periods of seed and inflorescence (the expression data value of two periods of each developmental stage all below 1 were excluded; Supplementary Table S6). Results found that 4 BdMAPKKK genes showed down-regulated expression and 6 BdMAPKKK genes showed up-regulated expression as the growth of inflorescence, of which *BdMAPKKK44, BdMAPKKK66*, and *BdMAPKKK29* changed expression more than 1.5-fold (log_2_-base value), while *BdMAPKKK7* gene was only expressed in emerging inflorescence, indicating these genes may take part in the regulation of inflorescence growth. Among development of seed after pollination, 9 BdMAPKKKs showed down-regulation expression, and 3 BdMAPKKKs showed up-regulation expression, of which the expression of *BdMAPKKK32, BdMAPKKK56, BdMAPKKK68* changed with fold more than 2 (log_2_-base value), and *BdMAPKKK10* gene was only expressed in seed 10 days after pollination, suggesting that they may be involved in the regulation of seed development. Overall, these results showed that the BdMAPKKK genes played multiple roles in the growth and development of *Brachypodium*.

To study the expression profiles of BdMAPKKs in three organs of *Brachypodium*, the SAM (Significant Analysis for Microarrays) method was used in MEV program. Significant differences of MAPKKK genes were analyzed with the *q*-value lower than 0.01, and 21 BdMAPKKK genes without ZIK members showed significant differences (Supplementary Table S7), including 5 MEKK and 16 Raf subfamily members, of which *BdMAPKKK16, BdMAPKKK31*, and *BdMAPKKK38* genes had higher expression in stem than that of other organs, implying that they may be participated in the signal transduction pathways in stems. Also, there 9 BdMAPKKK genes showing higher expression in leaves and 9 BdMAPKKK genes showing higher expression in roots were also found, respectively.

### Expression analysis of BdMAPKKK genes in virus, drought, and heat treatment

**Figure 7:**
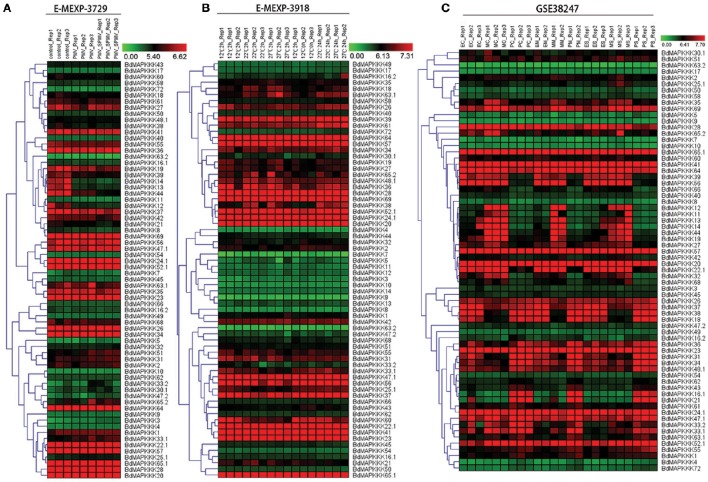
**The expression profile of MAPKKK genes in *Brachypodium* in virus, heat and drought stresses**. The red and green shading represents the relative high or low expression levels, respectively. The color bar represents log_2_ expression value. **(A)** The expression pattern of BdMAPKKK under virus treatment. **(B)** The expression pattern of BdMAPKKK under heat condition. **(C)** The expression pattern of BdMAPKKK for different level of drought treatment.

To reveal the functions of BdMAPKKK genes in the respond to viruses stress, we investigated the expression patterns of BdMAPKKKs after infecting with *Panicum mosaic virus* (PMV) and its satellite virus (SPMV) (Figure 7A). The differentially expressed genes defined as 1-fold change (log_2_-base ratio) with the *q*-value set with lower than 0.01. When the *Brachypodium* was infected only with PMV, there were only three BdMAPKKK genes (*BdMAPKKK12, BdMAPKKK13*, and *BdMAPKKK14*) showing significant differential expression compared with control and all of them were down-regulated (Supplementary Table S8-1). Simultaneously, when the *Brachypodium* was infected with PMV and SPMV together, additional two BdMAPKKK genes (*BdMAPKKK3* and *BdMAPKKK44*) showed significant differential expression, of which *BdMAPKKK3* was up-regulated (Supplementary Table S8-2). Taken together, a total of five BdMAPKKK genes responded to the viruses, of which four grouped into MEKK subfamily. These genes may be participated in the signal pathway of antiviral defense.

Crop plants are highly sensitive to ambient temperature, with a 1°C difference in temperature sufficient to affect development and yield (Boden et al., 2013). To study the roles of BdMAPKKK genes in response to high temperature stress, their expression patterns were investigated after the treatment with 12, 22, 27°C for 2 and 24 h (Figure 7B). The statistical significance of changes was assessed using two-way ANOVA method performed by MEV software with adjusted *p*-values threshold set 0.01 and the results were shown in Supplementary Table S8-3. When we set temperature condition as the main factor (adjusted *P* ≤ 0.01) and displaying 0.5-fold change (log_2_-base ratio)in 22, 27°C treatment compared with 12°C in any time, 3 BdMAPKKK genes showed significant differential expression in 22°C, of which 2 BdMAPKKK genes (*BdMAPKKK38* and *BdMAPKKK24*) were up-regulated and 1 BdMAPKKK gene (*BdMAPKKK44*) was down-regulated. Furthermore, a total of 8 BdMAPKKKs showed differential expression at 27°C, of which 6 BdMAPKKK genes were up-regulated and 2 BdMAPKKK genes (*BdMAPKKK44* and *BdMAPKKK60*) were down-regulated. Compared the members of differentially expressed genes under 22°C, all of the 3 significantly differentially expressed BdMAPKKK genes also differentially expressed in 27°C condition. When we set time condition as the main factor (adjusted *P* ≤ 0.01) and displaying 0.5-fold change (log_2_-base ratio) in 24 h treatment compared with 2 h in any temperature, the results showed only one BdMAPKKK gene (*BdMAPKKK37*) was related to the time of treatment, implying the most of BdMAPKKK genes were not significantly affected by the time of heat treatment. In addition, 4 BdMAPKKK genes (*BdMAPKKK16, BdMAPKKK20, BdMAPKKK58, BdMAPKKK64*) affected by the interaction of temperature and time of treatment (adjusted *P* ≤ 0.01) (Figure 7B). Promoter analysis showed there were two main elements involved in heat stress: HSE (AAAAAATTTC), TCA-element (GAGAAGAATA; Hasanuzzaman et al., 2013). The former was involved in heat stress responsiveness, and the latter involved in salicylic acid responsiveness. Most of BdMAPKKK genes responding to heat had HSE element or TCA-element and only two BdMAPKKK genes (*BdMAPKKK44* and *BdMAPKKK60*) were not, implying they may have certain unknown elements acting in roles in the heat defense (Supplementary Tables S8, S9).

Drought is one of the most serious stresses to plant growth, which could cause 45% reduction of the leaf size in *Brachypodium* (Verelst et al., 2013). To study the role of BdMAPKKK genes acting in three leaf zones under drought stress, their expression were analyzed after with moderate drought and severe drought treatment (Figure 7C). The differentially expressed genes were defined the fold change more than 1 (log_2_-base value) and 5 BdMAPKKK genes were identified (Supplementary Table S8-4). In the expansion region of leaf, 2 BdMAPKKK genes (*BdMAPKKK16* and *BdMAPKKK21*) were affected by moderate drought and when the condition was converted to the severe drought, additional one BdMAPKKK gene (*BdMAPKKK33*) expression was also significantly changed. No matter what the level of drought stress used, all the differentially expressed genes were down-regulated. In the leaf's mature zone, there were three BdMAPKKK genes (*BdMAPKKK16, BdMAAPKKK21*, and *BdMAPKKK30*) in responding to moderate drought stress but no BdMAPKKK genes to severe drought stress, whereas only *BdMAPKKK5* gene was regulated in proliferation zone of leaf under severe drought treatment (Figure 7C). Promoter analysis suggested there were four cis-acting regulatory elements related to drought stress, namely, ABRE (CCGCGTAGGC), motif II b (CCGCCGCGC), CGTCA-motif (CGTCA), and TGACG-motif (TGACG; Todaka et al., 2015). They were involved in abscisic acid (ABRE and motif II b) and MeJA-responsiveness (CGTCA-motif and TGACG-motif) which indicated they may act roles in the drought stress by regulating the abscisic acid or MeJA (Kuromori et al., 2014). All of the drought-responsive BdMAPKKKs had one or more drought related cis-elements, but only *BdMAPKKK16* gene were not found any these elements which may hold some other unknown elements playing roles in response to drought (Supplementary Table S9).

### Expression analysis of BdMAPKKK genes in diurnal conditions

**Figure 8:**
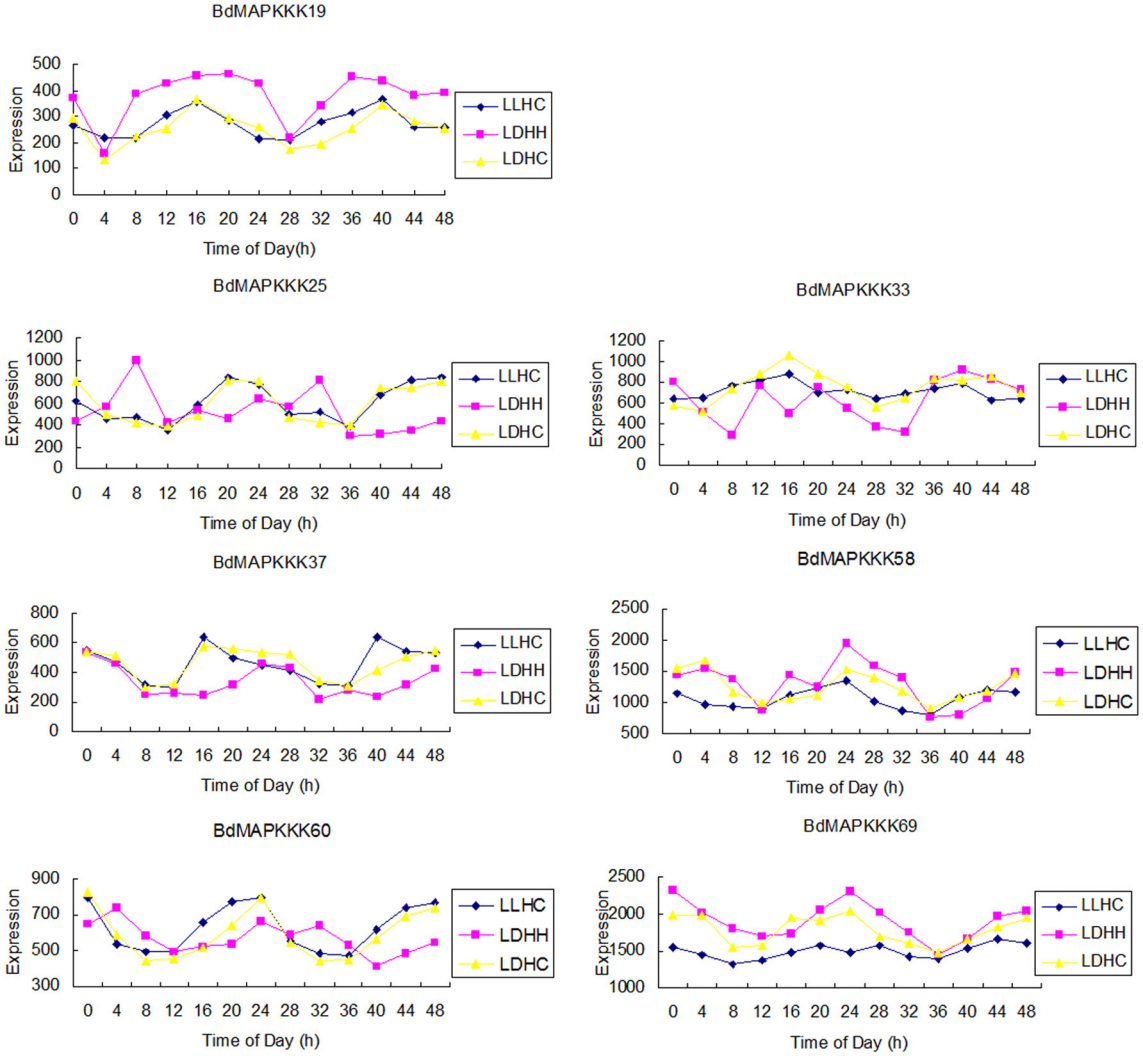
**The expression of MAPKKK genes cycled in three conditions**.

The circadian clock is an endogenous 24 h pacemaker that can anticipate the changes of environmental conditions and to coordinate the corresponding physiological and metabolic process by regulating the gene expression (Filichkin et al., 2011). In this study, the clock-associated BdMAPKKK genes were further identified using the Diurnal tool with the correlation cutoff value set 0.8. Results showed only 7 BdMAPKKK genes were expressed in all the three conditions studied (LDHH, Light day, Dark night, Hot day, Hot night; LDHC, Light day, Dark night, Hot day, Cold night; LLHC, Light day, Light night, Hot day, Cold night; Figure 8), and the detailed information were listed in Supplementary Table S10. Among them, 5 BdMAPKKK genes (*BdMAPKKK25, BdMAPKKK33, BdMAPKKK37, BdMAPKKK58, BdMAPKKK60)* were the members of Raf subfamily, and one gene for MEKK (*BdMAPKKK19*) and ZIK (*BdMAKKK69*) subfamily, respectively. The *AtWNK1* (*AtZIK4, At3g04910*) gene of *Arabidopsis* and *OsWNK1* (*OsMAPKKK20, LOC_Os07g38530*) of rice have been confirmed to play the roles in the regulation of daily rhythmicity (Murakami-Kojima et al., 2002; Nakamichi et al., 2002; Kumar et al., 2011), which provided the reliable evidence that the *BdMAPKKK69*, the orthologous gene of *AtWNK1* and *OsWNK1*, should also have the similar functions. Promoter analysis suggested that only *BdMAPKKK19* and *BdMAPKKK60* genes had circadian element(s) (CAANNNNATC) predicted. However, we found many of light responsive elements in the identified clock-associated genes, including sp1, G-box element, ACE, and 3-AF1 binding site element. The sp1 elements were all the identified BdMAPKKK genes possess, and G-box element was existed in 5 BdMAPKKKs, but ACE and 3-AF1 binding site element were only distributed in 2 BdMAPKKKs, implying the clock-associated BdMAPKKK genes cycling expression may be regulated by circadian or more than two different light responsive elements (Supplementary Table S9).

### Co-expression network of BdMAPKKKs, BdMKKs, and BdMPKs

With the development of gene array and RNA sequence technique, more and more transcript data could be acquired, which makes it possible to use bioinformatics to study the MAPK signal pathways. It has been demonstrated that similarity of expression patterns could indicate the correlation and biological function of genes (Eisen et al., 1998). Thus, co-expression network based on the correlations of gene expression levels is a useful method to investigate the gene interactions and regulatory relationship (Nayak et al., 2009; Obayashi and Kinoshita, 2009). In this study, we constructed the co-expression network of MAPK cascades in *Brachypodium* using 144 public available microarray and RNA-seq data based on the Mutual rank (MR) method.

**Figure 9:**
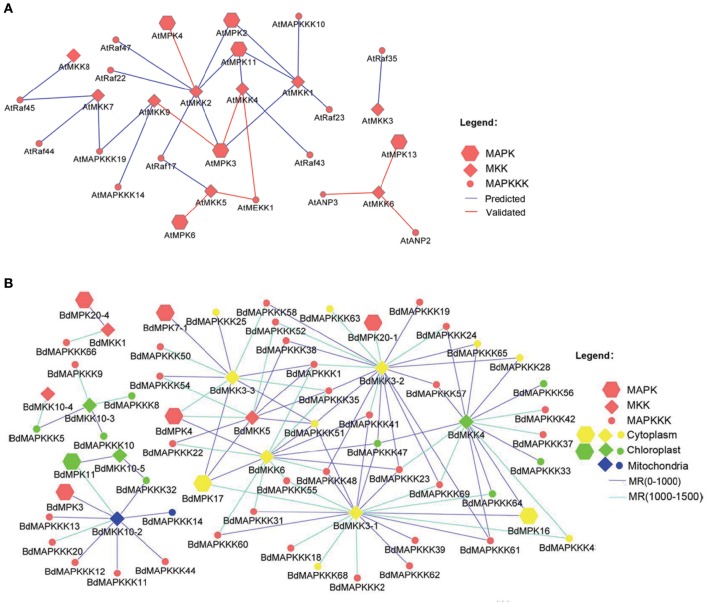
**The co-expression network of MAPKKK-MAPKK-MAPK gene family in *Arabidopsis* and *Brachypodium***. **(A)** The co-expression network of *Arabidopsis* and only the rank of MR value lower than 500 were shown. **(B)** The co-expression network of *Brachypodium* and only the MR value lower than 1500 were shown.

Considering that several MAPK signal pathway in Arabidopsis had been validated by various experiments, we firstly constructed the co-expression network of *Arabidopsis* MAPK cascades using the MR method to test the reliability of this method. A total of 4 previous reported signal pathway were identified, including 2 complete MAPKKK-MKK-MPK (*ANP2/3-MKK6-MPK13*; *MEKK1-MKK4/5-MPK3/6*) and two MKK-MPK (*MKK2-MPK4*; *MKK9-MPK3*) pathways (Figure 9A), suggesting the predicted MAPK signal pathways were reliable. Then, we used this method to predict the MAPK pathways with a relaxed MR value set 1000 and 1500 in Brachypodium (Figure 9B). The MR and weight PCC value information were listed in Supplementary Table S11. *BdMPK3, BdMPK4, BdMPK7-1, BdMPK16, BdMPK17, BdMPK20-1*, and *BdMPK20-4* genes belonging to BdMAPK family were predicted and each of them acted in multiple signal pathways. Also each of the BdMKK family members play roles in diverse pathways whereas the most of BdMAPKKKs only participated in one or two MAPK cascade, which indicated the MAPKKKs may control the whole cascade functions. When the standard set with the three main members in the predicted pathways showing the same subcelluar localization, only ten MAPK signal pathway were identified and most of were located in cytoplasm except for *BdMAPKKK32-BdMKK10-5-BdMPK11* predicted in chloroplast (Table 4). According to the gene expression analysis in different seed developments mentioned before, *BdMAPKKK32* gene was significantly down-regulated (log_2_-base value –10.73) and *BdMAPKKK68* was up-regulated (log_2_-base value 2.19), implying the three MAPK cascades *BdMAPKKK32-BdMKK10-5-BdMPK11* and *BdMAPKKK68-BdMKK3-1-BdMPK16/BdMPK17* may participate in the regulation of seed development. *BdMAPKKK25* gene showed a circadian expression in three conditions (LDHH, LDHC, and LLHC) illustrated above, which provided an indication *BdMAPKKK25-BdMKK3-3-BdMPK17* pathway may involve in daily rhythmicity. Possibly, the MAPKKK, MKK, and MPK also can be transferred into the same place to play interaction with each other. Although the data used in construction co-expression network was not comprehensive and may result in the false-positive ratio, we also get a glimpse of the regulatory network of MAPKs. This is the first study to construct the co-expression network of MAPK cascades in *Brachypodium* using the large scale of expression profiles data, which will provide an important foundation for us to study of MAPK transduction pathway in *Brachypodium*.

**Table 4:** **The predicted MAPK cascades with their three main members MAPKKK, MKK, and MPK with the same subcellular localization**.

**MAPK cascade**	**MAPKKK**	**MKK**	**MPK**	**Subcelluar localization**
1	BdMAPKKK32	BdMKK10-5	BdMPK11	Chloroplast
2	BdMAPKKK25	BdMKK3-3	BdMPK17	Cytoplasm
3	BdMAPKKK51	BdMKK3-3	BdMPK17	Cytoplasm
4	BdMAPKKK51	MKK6	BdMPK17	Cytoplasm
5	BdMAPKKK68	MKK3-1	BdMPK16	Cytoplasm
6	BdMAPKKK68	MKK3-1	BdMPK17	Cytoplasm
7	BdMAPKKK51	MKK3-1	BdMPK16	Cytoplasm
8	BdMAPKKK51	MKK3-1	BdMPK17	Cytoplasm
9	BdMAPKKK4	MKK3-1	BdMPK17	Cytoplasm
10	BdMAPKKK4	MKK3-1	BdMPK16	Cytoplasm

## Conclusions

MAPK cascades as a highly conserved signal transduction module in eukaryotes, play the important roles in the development and stress responses in plant. In this study, we identified 86 MAPKKKs proteins encoded by 73 MAPKKK genes in *Brachypodium*. Phylogenetic study of MAPKKKs family from *Arabidopsis*, rice, and *Brachypodium* have classified them into three subfamily, of which 28 were MEKK, 52 were Raf and 6 were ZIK subfamily, respectively. In each subfamily, the protein motif and exon-intron organization and splicing intron phase in kinase domain were conserved, which supported the phylogenetic analysis. To explore the evolution patterns of MAPKKK, we calculated the Ka/Ks value and all of them were lower than 1, which indicated the MAPKKK genes were under strong purifying selection. Furthermore, the expression patterns of BdMAPKKK genes in different organs and development stages as well as heat, virus and drought stresses were investigated to identify the developed-specific or stress-responsive BdMAPKKK genes, which provided the candidates for further functional analysis. Additionally, the BdMAPKKK genes related to circadian were also characterized. Finally, co-expression network of three subfamily members was constructed, and we predicted the 10 potential MAPK cascade pathways in *Brachypodium*. This study systematically reported the structure, evolutionary, and expression as well as the co-expression network features of BdMAPKKK gene family, which will provide important information for further functional analysis of BdMAPKKKs, and also will contribute to better understanding the molecular mechanism of development and stresses signal transduction in *Brachypodium* and beyond.

## Author contributions

WT and XN conceived and designed the experiments, KF, FL, and JZ, performed the gene identification and structural research, GX and PD analyzed the expression data, XN and WS provided project resources, WT and XN wrote the manuscript. All authors read and approved the final manuscript.

## Funding

This work was mainly funded by the National Natural Science Foundation of China (Grant No. 31401373) and partially supported by the Open Project Program of State Key Laboratory of Crop Stress Biology in Arid Areas, China (CSBAA2014002).

### Conflict of interest statement

The authors declare that the research was conducted in the absence of any commercial or financial relationships that could be construed as a potential conflict of interest.
